# Non-specific symptoms-based pathways for diagnosing less common cancers in primary care: a service evaluation

**DOI:** 10.3399/BJGP.2020.1108

**Published:** 2021-09-21

**Authors:** Dave Chapman, Veronique Poirier, Karen Fitzgerald, Brian D Nicholson, Willie Hamilton

**Affiliations:** Cancer Research UK, London.; Cancer Research UK, London.; Cancer Research UK, London.; Nuffield Department of Primary Care Health Sciences, University of Oxford, Oxford.; College of Medicine and Health, University of Exeter Medical School, Exeter.

**Keywords:** neoplasms, less common cancers, Multidisciplinary Diagnostic Centre, non-specific symptoms, primary health care, referral

## Abstract

**Background:**

Although less common cancers account for almost half of all cancer diagnoses in England, their relative scarcity and complex presentation, often with non-specific symptoms, means that patients often experience multiple primary care consultations, long times to diagnosis, and poor clinical outcomes. An urgent referral pathway for non-specific symptoms, the Multidisciplinary Diagnostic Centre (MDC), may address this problem.

**Aim:**

To examine the less common cancers identified during the MDC pilots and consider whether such an approach improves the diagnosis of these cancers.

**Design and setting:**

A service evaluation of five MDC pilot projects in England from December 2016 to March 2019.

**Method:**

Data items were collected by pilot sites in near-real time, based mainly on the English cancer outcomes and services dataset, with additional project-specific items. Simple descriptive and comparative statistics were used, including χ^2^ tests for proportions and *t*-tests for means where appropriate.

**Results:**

From 5134 referrals, 378 cancers were diagnosed, of which 218 (58%) were less common. More than 30 different less common tumour types were diagnosed in this cohort. Of the MDC patients with less common cancers, 23% (*n* = 50) had ≥3 GP consultations before referral and, at programme level, a median time of 57 days was recorded from GP urgent referral to treatment for these tumour types.

**Conclusion:**

A non-specific symptomatic referral route diagnoses a broad range of less common cancers, and can support primary care case management for patients with symptoms of possible cancer that do not qualify for a site-specific urgent referral.

## INTRODUCTION

Rare and less common cancers (hereafter ‘less common cancers’) account for almost half of all cancer diagnoses in England and over half of all cancer deaths.^1^^–^^3^ This broad term incorporates >200 different tumour types, excluding the four most common malignancies: breast, colorectal, lung, and prostate cancers (hereafter ‘common cancers’).^4^

With the exception of cervical cancer, there is currently no established screening programme for less common cancers,^5^ and recognition of disease relies on the development and presentation of symptoms.^6^^–^^8^ In many cases, these cancers present with non-specific symptoms, which can also originate from multiple benign conditions.^6^^,^^9^^–^^11^ For example, unexpected weight loss is associated with several cancers at all cancer stages but may also arise from serious and non-serious diagnoses associated with a wide range of body systems.^12^^–^^13^ Additionally, the relative scarcity of less common cancers often makes the risk of cancer in symptomatic patients lower than the UK’s recommended 3% threshold for urgent cancer investigation, even when symptoms are highly specific to the cancer.^5^^,^^6^^,^^9^^,^^14^ The range of possible conditions and the low likelihood of cancer complicates the choice and timing of diagnostic investigation in primary care.

The diagnostic process for patients diagnosed with less common cancers as well as those presenting with nonspecific symptoms is often characterised by multiple primary care consultations, investigations, and referrals.^15^^–^^19^ Lengthy intervals from presentation to diagnosis are common,^6^^,^^16^^,^^17^^,^^20^ as is diagnosis by emergency presentation,^16^^,^^21^^–^^22^ with both being associated with high rates of advanced stage diagnosis,^16^ worse survival,^23^ and a poorer experience of care.^3^^,^^24^

A Multidisciplinary Diagnostic Centre (MDC) approach was piloted in England from December 2016, establishing a dedicated pathway for patients presenting with nonspecific symptoms indicative of possible cancer. An evaluation by the Accelerate Coordinate Evaluate (ACE) Programme, which aimed to improve cancer pathways and associated outcomes through the provision of evidence-based information and support,^25^ demonstrated that the MDC approach diagnosed a broad range of cancers, including a notable proportion of less common cancers.^10^ The aim of this study was to examine the less common cancers identified during the MDC pilots in detail and to consider whether such an approach has benefit for the diagnosis of these cancers.

## METHOD

### MDC projects

The ACE Programme evaluation comprised five projects in England, incorporating 10 operational MDC pilot sites (one in Airedale, two in Greater Manchester, one in Leeds, five in London, and one in Oxford).^10^

**Table:** How this fits in

Almost half of all cancer diagnoses in England are of less common cancer types, many of which can be characterised by non-specific symptoms presentation, long diagnostic intervals and poor clinical outcomes. Five Multidisciplinary Diagnostic Centres (MDCs) were piloted across 10 English sites as a rapid referral route for the investigation of primary care patients with non-specific cancer symptoms. Most cancers diagnosed by the MDCs were ‘less common’ cancers comprising >30 different tumour types. These cancers typically have long diagnostic intervals and have poor clinical outcomes. The broad range of less common cancers diagnosed rapidly by MDCs emphasises the value of diagnostic pathways that aim to establish the cause of symptoms instead of ruling out individual tumour types.

Projects were established to assess a dedicated urgent referral route for patients presenting with a predetermined range of non-specific symptoms for which there was no clear diagnostic approach. The pathway predominantly offered a single referral route for primary care, although a number of projects also allowed a smaller volume of referrals from other agencies.^10^^,^^26^

Individual hospital sites were launched at different times from December 2016 to January 2018. To reflect the evaluation’s design, programme-funded activity with the MDC pilots concluded on 31 March 2019.

### Referral criteria

MDC project referrals were limited to adult patients aged ≥18 years (in Oxford ≥40 years), presenting with non-specific but concerning symptoms, such as unexplained weight loss, non-specific pain, unexplained appetite loss, and persistent fatigue. Eligibility criteria varied at project level (see Supplementary Box S1 for details), but all projects focused exclusively on patients of clinical concern whose nonspecific symptoms were potentially indicative of cancer or other serious disease.^10^^,^^26^ To be eligible for referral, the patient’s symptoms also had to be insufficiently clear to identify an appropriate tumour-specific urgent referral pathway. Patients with previous cancers were considered eligible for referral, provided that they had non-specific symptoms only.

### Data collection and analysis

A programme dataset was agreed for all MDC projects to ensure a uniform approach to data collection. Data items were based mainly on the English cancer outcomes and services dataset,^27^ with additional project-specific items focusing on secondary care presentation, diagnostic process of cancers, and other diseases. Data management arrangements varied by MDC project, and used a combination of local healthcare IT systems and bespoke data systems, with data items collected as close to real-time as possible.^10^ Minor recoding was applied by programme evaluators to align the data for analysis.

Simple descriptive and comparative statistics were used, including χ^2^ tests for proportions and *t*-tests for means where appropriate, which concentrate on diagnoses of less common cancers within this referral cohort. These have been aggregated to a programme level to provide greater scope for analysis. No formal power calculation was made relating to the expected cancer yield.

This study on less common cancers, which covers MDC pathway activity from December 2016 to March 2019, is one of several pathway analyses and contributes to existing evidence on initial MDC results.^10^ Further analyses are planned on MDC diagnostic activity and will consider the overall use of computed tomography as a diagnostic investigation and any impact on pathway time, in addition to the pathway’s diagnosis of non-cancer disease.

Although common cancers have dedicated referral pathways in place,^9^ and often present with recognised high-risk site-specific symptoms, patients with these cancers may experience nonspecific symptoms, and thus enter the MDC pathway. As the pathway aims to provide a route to diagnosis for symptomatic patients whose cancer is indistinguishable at point of presentation, data on the presentation of common cancers have been included to reflect the difficulty facing the referrer.

A list of symptoms was identified in the dataset and developed with clinical guidance to describe patients whose presentation was suggestive of cancer but did not indicate a specific diagnostic approach.^10^ This range of symptoms included some conditions and signs that were not strictly symptoms (see Supplementary Box S2 for details).

## RESULTS

From December 2016 to March 2019, 5134 patients were referred to the pilot MDCs. Of a total 378 cancers diagnosed, 218 (58%) were less common cancers (Table 1); therefore, the MDC pathway recorded an overall conversion rate of 7%, with 4% of the cancers diagnosed being of less common cancer types. The most common diagnoses related to upper gastrointestinal (39%), haematological (25%), and urological (14%) cancers. For five cancers, a confirmed diagnosis was recorded but without additional information on tumour site; these five cancers were excluded from analyses as details of their final diagnoses were unavailable.

**Table 1.** Anatomical sites of less common cancers diagnosed in the Multidisciplinary Diagnostic Centre

**Tumour group, *n* (%)**	**Tumour description (ICD 10 code)**	***n***
Upper GI, 84 (39)	Malignant neoplasm of pancreas (C25)	43
	Malignant neoplasm of stomach (C16)	11
	Malignant neoplasm of liver and intrahepatic bile ducts (C22)	11
	Malignant neoplasm of oesophagus (C15)	9
	Malignant neoplasm of gallbladder (C23)	6
	Malignant neoplasm of other and unspecified parts of biliary tract (C24)	3
	Malignant neoplasm of other and ill-defined digestive organs (C26)	1
Haematological, 54 (25)	Non-Hodgkin’s lymphoma (C82–C86, C96)	32
	Multiple myeloma and malignant plasma cell neoplasms (C88, C90)	12
	Hodgkin’s disease (C81)	5
	Acute myeloblastic leukaemia (C92–C95)	1
	Other leukaemia of specified cell type (C94)	3
	Lymphoid leukaemia (C91)	1
Urological, 31 (14)	Malignant neoplasm of kidney, except renal pelvis (C64)	25
	Malignant neoplasm of bladder (C67)	5
	Malignant neoplasm of renal pelvis (C65)	1
Other, 18 (8)	Malignant neoplasm without specification of site (C80)	15
	Malignant neoplasm of adrenal gland (C74)	1
	Malignant neoplasm of other and ill-defined sites (C76)	1
	Secondary malignant neoplasm of other sites (C79)	1
Gynaecological, 9 (4)	Malignant neoplasm of ovary (C56, C57)	8
	Malignant neoplasm of uterus, part unspecified (C55)	1
Sarcoma, 9 (4)	Malignant neoplasm of retroperitoneum and peritoneum (C48)	7
	Malignant neoplasm of pelvic bones, sacrum, and coccyx (C41)	1
	Malignant neoplasm of other connective and soft tissue (C49)	1
Skin, 5 (2)	Malignant melanoma of the skin (C43)	5
Lung/pleura, 4 (2)	Mesothelioma (C45)	3
	Malignant neoplasm of thymus (C37)	1
Head & neck, 2 (1)	Malignant neoplasm of dorsal surface of tongue (C02)	1
	Malignant neoplasm of mouth (C06)	1
Lower GI, 1 (0.5)	Malignant neoplasm of small intestine (C17)	1
Brain/CNS, 1 (0.5)	Malignant neoplasm of cerebrum, brain, unspecified (C71)	1
**Total cancers**		**218**

*CNS = central nervous system. GI = gastrointestinal. ICD 10 = International Classification of Diseases, 10th Revision.*

In addition to cancers diagnosed, 2060 patients were diagnosed with at least one non-cancer condition. Based on the overall number of diseases recorded (*n* = 2383), most cases (*n* = 989; 42%) related to conditions of the digestive system, including gastritis and duodenitis, and diverticular disease. A variety of other non-cancer disease was evident, including conditions relating to abnormal clinical and laboratory findings (*n* = 279; 12%) (mainly abnormal findings on diagnostic imaging of the lungs), and respiratory disease (*n* = 170; 7%), including diagnoses of bronchiectasis, emphysema, and other interstitial pulmonary disease. Diseases of the genitourinary system (*n* = 166; 7%) were also diagnosed, as were several types of benign neoplasm (*n* = 130; 5%) (data not shown).

Table 2 describes the age and presenting features of patients diagnosed with cancer in the MDC. Symptoms accounting for <5% of symptoms overall have been grouped and classified as ‘other’.

**Table 2.** Presenting features of Multidisciplinary Diagnostic Centre patients by cancer type

	**Less common cancers (all)**		**Common cancers**	

	***n***	**%**	***n***	**%**
**Patient age range, years**				
<50	7	3	5	3
50–75	114	52	78	50
>75	97	44	72	46
All cases	218	—	155	—

**Presenting feature**				
Weight loss	132	26	99	27
GP ‘clinical suspicion’	90	17	74	20
Nausea/appetite loss	71	14	52	14
Pain	58	11	40	11
Fatigue	47	9	39	10
Abnormal test results (for example, bloods or urine)	30	6	26	7
Anaemia	29	6	14	4
‘Other’ symptoms (with <5% instances)^a^	59	11	29	8

**Total symptoms recorded**	516	100	373	100

Presentation with ≥2 symptoms (including GP ‘clinical suspicion’), *n/N* (%)	148/218 (68)		109/155 (70)	

Patients with ≥3 GP consultations before referral based on available records, *n/N* (%)	27/116 (23)		25/90 (28)	

a *Other symptoms include: patient/family concern; general condition; respiratory problem; jaundice; bloating; change in bowel habit; lymphadenopathy; thrombocytosis; hypercalcaemia; and deep vein thrombosis. Despite being considered a site-specific symptom, jaundice was included as a referral criterion in London MDC to reflect locally determined clinical priorities. MDC = Multidisciplinary Diagnostic Centre.*

Patients diagnosed with less common cancers had a median age of 74 years (range 30–93 years) (data not shown). The most common reasons for referral to the MDC for less common and for common cancers, respectively, were: weight loss (26%; 27%), GP ‘clinical suspicion’ (17%; 20%), nausea/appetite loss (14%; 14%), and pain (11%; 11%) (Table 2). However, there was no difference in the association between the reason for referral and a diagnosis of a common or less common cancer (χ^2^ = 0.19, with 7 degrees of freedom). Most patients (68%) diagnosed with less common cancers presented with ≥2 non-specific symptoms, with the most common pairings being ‘weight loss and nausea’ (*n* = 57), and ‘weight loss and GP ‘clinical suspicion’ (*n* = 57) (data not shown). Based on 210 completed patient records, 25% of patients overall had ≥3 consultations with their GP before referral (23% less common; 28% common) (Table 2).

Table 3 describes the presenting features of patients diagnosed with the three most frequently diagnosed less common cancers: kidney cancer, non-Hodgkin’s lymphoma, and pancreatic cancer.

**Table 3.** Presenting features of Multidisciplinary Diagnostic Centre patients diagnosed with kidney, non-Hodgkin’s lymphoma, and pancreatic cancers

	**Kidney (C64)**		**Non-Hodgkin’s lymphoma (C82–C86, C96)**		**Pancreas (C25)**	

	***n***	**%**	***n***	**%**	***n***	**%**
**Patient age range, years**						
<50	—	—	—	—	—	—
50–75	16	64	16	50	22	51
>75	9	36	16	50	21	49
All cases	25	100	32	100	43	100

**Presenting feature**						
Weight loss	18	33	20	25	29	26
GP ‘clinical suspicion’	5	9	17	21	18	16
Nausea/appetite loss	7	13	11	14	19	17
Pain	5	9	8	10	16	14
Fatigue	7	13	5	6	9	8
Abnormal test results (for example, bloods or urine)	3	6	6	7	8	7
Anaemia	5	9	6	7	3	3
‘Other’ symptoms (with <5% instances overall)^a^	4	7	8	10	10	9

**Total symptoms recorded**	54	100	81	100	112	100

Presentation with ≥2 symptoms (including GP ‘clinical suspicion’), *n/N* (%)	17/25 (68)		24/32 (75)		30/43 (70)	

a *Other symptoms include: patient/family concern; general condition; respiratory problem; jaundice; bloating; change in bowel habit; lymphadenopathy; thrombocytosis; hypercalcaemia; and deep vein thrombosis. Despite being considered a site-specific symptom, jaundice was included as a referral criterion in London MDC to reflect locally determined clinical priorities. MDC = Multidisciplinary Diagnostic Centre.*

Variation was noted at a tumour-specific level, with kidney cancers more commonly diagnosed in patients aged ≤75 years, having also presented with higher proportions of weight loss (33%) and fatigue (13%), in addition to a lower proportion of GP ‘clinical suspicion’ (9%) (Table 3). The proportion of GP ‘clinical suspicion’ was highest for diagnoses of non-Hodgkin’s lymphoma (21%), and rates of nausea/appetite loss were higher in pancreatic cancers (17%). Non-Hodgkin’s lymphoma was associated with higher rates of presentation with ≥2 non-specific symptoms. Because of small sample sizes; however, this information is provided for descriptive purposes only.

Table 4 provides details of the duration from GP urgent referral to the start of any cancer treatment. Data on interval times from GP urgent referral to start of cancer treatment were only available for 135 of 218 (62%) less common cancer diagnoses. Interval times have been provided at tumour site level in cases where there was a sufficient number of cases to support analysis. In some instances, the number of cases was too small to calculate the centiles reliably. Rates have been provided for median, interquartile range, and 90% centile relating to the pathway’s treatment interval. As these figures respectively include and compensate for outlier records, and provide a figure representative of 90% of the pathway’s activity, they collectively provide a balanced and robust representation of pathway time to cancer treatment.

**Table 4.** Reported interval time in the Multidisciplinary Diagnostic Centre from GP urgent referral to start of any cancer treatment

	**Median, days**	***n***	**IQR**	**90% centile**	**% ≤62 days**
**Less common cancers (all cases)^a^**	**57**	**135**	**34–78**	**111**	**54**
Gynaecology	50	6	26–71	79	50
Haematology	65	34	26–88	111	44
Other	39	9	21–49	65	78
Sarcoma	41	5	36–44	55	80
Upper GI tract	47	52	33–67	93	63
Urology	74	21	48–110	163	33
Haematology: NHL only	63	25	27–90	108	44
Upper GI tract: pancreas only	45	31	32–63	82	71
Upper GI tract: oesophago-gastric	46	12	31–62	68	75
Urology: kidney only	74	17	48–110	144	35
‘Selected’ cancer sites^b^	49	109	29–71	97	60

a *At tumour group level, cancers with <5 diagnoses have been excluded.*

b *Selected cancer sites are defined as having ICD 10 codes that are not breast, lower GI, lung, skin, or urological. The cohort has been constructed to reflect available data published as part of national cancer statistics (where it is referred to as ‘other’). GI = gastrointestinal. ICD 10 = International Classification of Diseases, 10th Revision. IQR = interquartile range. NHL = non-Hodgkin’s lymphoma.*

Table 5 shows the stage distribution of cancers diagnosed in the MDC, and indicates that most diagnoses were of a late stage (III/IV) (79%). However, a notable proportion of early-stage diagnoses were recorded for these cancers, including for kidney cancers (29%).

**Table 5.** Stage distributions of less common cancers diagnosed in the Multidisciplinary Diagnostic Centre

	**Less common cancers (all), *n* (%)**	**Kidney (C64), *n* (%)**	**Non-Hodgkin’s lymphoma (C82–C86, C96), *n* (%)**	**Pancreas (C25), *n* (%)**
Early stage (I/II)	31 (21)	5 (29)	4 (24)	8 (23)
Late stage (III/IV)	120 (79)	12 (71)	13 (76)	27 (77)
Unknown	65	8	15	8
Incomplete	2	—	—	—
Subtotal	151	17	17	35
Total	218	25	32	43

## DISCUSSION

### Summary

This study has demonstrated that a dedicated urgent referral pathway focusing on non-specific symptoms rapidly identified a broad range of less common cancers, with >30 different tumour types detected from a total of 218 less common cancers. The MDC pathway recorded an overall cancer conversion rate of 7%, with over half the diagnoses being of less common cancers (an identification rate of 4% for these cancers). An MDC referral therefore selects a population with an overall cancer positive predictive value exceeding the 3% recommended for urgent investigation.^9^ Crucially, it provides a pathway for the diagnosis of rarer cancers that individually fall beneath this 3% threshold, but are collectively above it. MDC referral can therefore support primary care case management for patients with symptoms of possible cancer that do not qualify for an urgent site-specific referral.

### Strengths and limitations

This study has several limitations. The MDC provides a referral route for a new cohort of patients, for whom a direct comparator is not available, and this study examines the diagnosis of less common cancers, for which national statistical information is less complete than for common cancers. Both of these factors have tempered judgements on the MDC’s possible impact compared with existing pathways for individual less common cancers. Analyses in this study have also been restricted by the relatively small numbers of some less common cancers, although this is a challenge common to diagnostic studies of any uncommon disease.^28^^–^^29^ Although presentation with non-specific symptoms, both individually and in combination, has been considered, it has not been possible to ascribe any significance to these analyses as a result of the level of bias introduced into the study by the establishment of the pathway’s referral criteria. Therefore, while the presence of these symptoms will be heightened in the study, it is not possible to extrapolate this to a wider population.

A direct comparison with national 62-day wait performance was hampered by a lack of published data at tumour site level, and by the MDC’s unique focus on non-specific symptoms as a patient cohort, meaning that a viable comparator for the pathway’s treatment interval could not be established. Because of the time-limited nature of the evaluation, judgements regarding the longer-term impact of the pathway on patient outcomes have not been possible, but further research into this area would be of great value. Finally, the study focuses on the results of a service evaluation and, although measures were established to support data collection and reporting, some variation was noted in data completeness and in the interpretation of some data items at project level. In cases where variation and/or incomplete recording was noted, for example, performance status and comorbidity, data items were excluded from the study.

### Comparison with existing literature

Other studies have examined the presenting features and diagnostic pathways of several less common cancers.^6^^,^^20^ Many of these have indicated that less common cancers are subject to long diagnostic intervals and have poor clinical outcomes regarding stage and mortality.^8^^,^^20^^,^^22^^,^^30^ Similarly, some recent studies have considered the merits of diagnostic pathways for non-specific symptoms,^10^^,^^16^ albeit with a wider focus on cancer overall. The current study adds to this body of evidence by considering the impact of non-site-specific symptomatic referral on the diagnosis of a broad range of less common cancers.

### Implications for research and practice

The challenge facing primary care in diagnosing less common cancers presenting with non-specific symptoms is well documented. As an urgent referral pathway for non-specific symptoms, the MDC diagnosed a higher proportion of less common cancers than common cancers (*n* = 218 versus *n* = 155). This was anticipated because presentation with nonspecific symptoms is considered normal for several less common cancers, including some of those frequently diagnosed in the MDC; for example, upper gastrointestinal (20% non-specific, 7% characteristic) and haematology (12% non-specific, 8% characteristic), compared with breast and prostate cancer (1% non-specific, 16% characteristic; 10% non-specific, 22% characteristic, respectively, although latter figures relate to urological cancers as a whole).^16^

In this study, 25% of patients in the MDC had ≥3 GP consultations before referral. This figure is below the equivalent rate of 32% for patients with non-specific symptoms reported in the National Cancer Diagnosis Audit,^16^ and is suggestive of a reduction in pre-referral consultation activity for this cohort. As the number of consultations before referral has been shown to have validity as a measure of the wider primary care interval,^30^ it is arguable that such a reduction via the MDC pathway could support faster diagnosis for some less common cancers, and for patients with non-specific symptoms overall.

In addition to cancer diagnoses, >2000 patients were diagnosed with at least one non-cancer condition, with these diagnoses representative of a broad range of non-malignant disease. As non-specific symptoms can potentially stem from multiple benign and/or serious conditions, the MDC’s focus on resolving symptoms rather than ruling out specific disease enables such a spectrum of diagnoses to occur. This approach can also support connectivity across the surrounding healthcare system through informed onward referral at point of diagnosis within the MDC, and may have benefit regarding ongoing patient surveillance for certain diagnosed conditions.

At a programme level, a median time of 57 days from GP urgent referral to treatment was recorded for less common cancers in the MDC, which is in line with the national 62-day wait standard.^31^ Treatment intervals varied by tumour site, with notably shorter median intervals reported for sarcoma, upper gastrointestinal, and ‘other’ cancers, but longer intervals identified for haematology and urology. A partial comparison against national 62-day wait compliance suggests the MDC is faster for oesophago-gastric cancers (75% MDC; 71% England),^32^ but moderately slower for other ‘selected’ cancer sites (60% MDC; 69% England).^33^ As the study relates to activity in pilot sites, it is plausible that these interval times may improve as the pathway matures and becomes fully embedded and resourced. Cumulatively, by reducing prereferral activity and providing a swift overall time to treatment, the MDC may also lessen the chance of diagnosis via emergency presentation, which is common for both non-specific symptoms,^16^ and many less common cancers such as kidney,^14^^,^^21^^,^^34^^–^^35^ non-Hodgkin’s lymphoma,^21^^,^^34^ and pancreatic cancer.^21^^,^^34^^,^^36^

Most cancers in the MDC were diagnosed at a late stage, which may reflect the systemic nature of some of the non-specific symptoms eligible for MDC referral. Even so, 21% of less common cancers were diagnosed at stage I/II, with rates varying by tumour site but limited by insufficient numbers in some instances. Where site-specific data were available, early-stage diagnosis for pancreatic cancer was consistent with the national rate (23% MDC; 23% England),^37^ suggesting that the pathway may offer benefit for some tumour sites with very poor early-stage diagnosis. The proportion of early-stage diagnosis was lower than the national rate for cases of non-Hodgkin’s lymphoma (24% MDC; 30% England),^37^ and kidney cancer (29% MDC; 57% England).^37^ However, when interpreting this information, it is necessary to consider the strong association between non-specific symptoms and late-stage diagnosis. As the MDC focuses exclusively on this patient cohort, it will be disadvantaged in any comparison with national figures, within which this patient cohort will not be visible.

To gain a more comprehensive understanding of the MDC’s impact on pathway interval times and variation among differing diagnoses, further research, and the publication of cancer waiting time data for less common cancers will be required. It is important to gauge whether any benefits gained by faster times to diagnosis lead to earlier initiation of cancer treatment. Such work should also consider how current system capacity and access to specialist treatment may affect interval times, particularly given the specialist requirements for some rarer cancers. Further pathway evaluation is also merited on the balance of benefit and harm associated with diagnostic investigation, as only a minority of referrals with possible malignancy actually result in a cancer diagnosis. This additional information would contribute to the evidence base regarding non-specific symptoms and rarer cancers, and would directly inform the development and implementation of the rapid diagnostic centre model in England,^38^ which has evolved from the MDC approach. As these new pathways are established throughout England, it will be important to retain a focus on non-specific symptoms as a distinct cohort of patients, in order to build on the potential demonstrated in the MDC pilots. Such an approach may also offer an opportunity to develop a dedicated, integrated diagnostic interface between primary and secondary care, in support of the swift recognition, referral, and diagnosis of cancers presenting with non-specific symptoms.
